# Supplementary material for: Violence against older women: A systematic review of qualitative literature
Source: PLoS One. 2020 Sep 24;15(9):e0239560. doi: 10.1371/journal.pone.0239560 (PMC7514024; doi:10.1371/journal.pone.0239560)
Supplement: Attachment — Submitted filename: 2020 Plos One review Sarah.docx [file pone.0239560.s004.docx]

Thank you for giving me the chance to review this important manuscript that addresses a clear gap in the literature. It is very clear that you have done an amazing job in thoroughly going through a vat amount of literature and put a lot of attention in capturing necessary detail. Congratulations on it!

I have some general and specific comments.

General comments:

- The title of the paper is violence against older women and throughout the text you refer to older women. The definition of older women in the text is women aged 50 or older. Is this aligned with existing definition of older or is it a consequence that many other studies have focused on women of reproductive age? Should you not throughout the title and text to simply name them women aged 50 or older or is older women the correct term?
- The review stats that it is “exploring violence against women aged 50 and above, identifying types and patterns of violence, perpetrators of violence, and impacts of violence on various health outcomes for older women”. Reading the review, it seems that much more has been investigated than that, namely associate factors and consequences beyond health outcomes, such as loneliness and social isolation. This could be stated more clearly in the introduction, as I kind of expected it but did not find it reflected in the description of the reviews scope.
- Throughout the results section it was often unclear whether you are summarising violence described actual violence that older women are experiencing now or whether they refer to any violence as it sounds like in the section on loneliness (not only an issue there)? It is important to make that very clear whether women refer to past violence, potentially 20 years ago or current violence. For example, but not only there, in the section “Silence, stigma and family” it is unclear if the women refer to the current violence they experienced or past violence, potentially 20 years ago. The whole paragraph seems to refer to varies time points in which the violence happened and this needs to be clarified, especially since there is a specific sub-section for it
- Have you actually found any differing evidence by different age categories among women aged 50 or above? In the limitation section you mention that the age rages varied widely, but it would be could to situate the results into this as 50 to 64 is quite a different age category than 65 to 99, when women are also more likely to be retired.
- I am a bit surprised by the structuring of the results section and consider reworking it as it jumps from the overview of forms of violence and perpetrators in a summary paragraph to causes, consequences, risk factors to financial abuse- a form of violence experienced, norms, needs, early childhood violence as a risk factor later. While the overall heading make sense, the sub heading sometimes seem to belong somewhere else and do not flow.
- Did you actually find different results by LMIC and HIC?
- The section “Descriptions of types of violence and perpetrators” is actually a quite crucial one, but it is currently very descriptive in terms of numbers. Given that forms of violence and perpetrators are such a key objective, could you expand this section and show what kind of violence was perpetrated by the different perpetrator types or forms of abuse and whether there were any age trends among the older women.

Small, specific comments:

- Abstract p 8, line 31: grammatical mistake

Introduction:

- Page 4, line 77 needs a reference
- Page 6, line 116: Can you state if these systematic reviews said anything about the perpetrators of this violence? Which age ranges did the systematic reviews investigate?
- Page 6, line 128 grammatical error

Method:

- Did you use any time limit for the search?
- Did forms/types of violence cluster in certain countries?

Results:

- On page 14 under the heading “Descriptions of types of violence and perpetrators” you first describe the terms IPV, family violence and elder abuse, but these terms have not been described before in terms of what they mean and how they are different from each other
- On page 15 when you talk about causes of violence, which types of violence were referred to?
- Is the financial abuse section actually referring to tis as a form of violence or a cause for other violence or a co-occurrence of numerous forms of violence?
- In the discussion section you refer to health care providers response and women’s concerns regarding confidentiality, however, this was not brought up in the results at all and should have been mentioned there too.
- Implication for future research – the scarcity of research only relates to qualitative work or more generally? I understand that your review mainly found studies on IPV, but what did the quantitative reviews find? Would they support your claim?
